# Improved Gait of Persons With Multiple Sclerosis After Rehabilitation: Effects on Lower Limb Muscle Synergies, Push-Off, and Toe-Clearance

**DOI:** 10.3389/fneur.2020.00668

**Published:** 2020-07-24

**Authors:** Johanna Jonsdottir, Tiziana Lencioni, Elisa Gervasoni, Alessandro Crippa, Denise Anastasi, Ilaria Carpinella, Marco Rovaris, Davide Cattaneo, Maurizio Ferrarin

**Affiliations:** IRCCS Fondazione Don Carlo Gnocchi, Milan, Italy

**Keywords:** muscle synergies, multiple scleorsis (MS), rehabilitation, gait, EMG, push-off, toe-clearance

## Abstract

**Introduction:** Persons with MS (PwMS) have markedly reduced push-off and toe-clearance during gait compared to healthy subjects (HS). These deficits may result from alterations in neuromotor control at the ankle. To optimize rehabilitation interventions for PwMS, a crucial step is to evaluate if and how altered neuromotor control, as represented by muscle synergies, improves with rehabilitation. In this study we investigated changes in ankle motor control and associated biomechanical parameters during gait in PwMS, occurring with increase in speed after gait rehabilitation.

**Methods:** 3D motion and EMG data were collected while 11 PwMS (age 50.3 + 11.1; EDSS 5.2 + 1.2) walked overground at self-selected speed before (T0) and after 20 sessions (T1) of intensive treadmill training. Muscle synergies were extracted using non-negative matrix factorization. Gait parameters were computed according to the LAMB protocol. Pearson's correlation coefficient was used to evaluate the similarity of motor modules between PwMS and HS. To assess differences in distal module activations representing neuromotor control at the ankle [Forward Propulsion (FPM) and Ground Clearance modules (GCM)], each module's activation timing was integrated over 100% of the gait cycle and the activation percentage index (API) was computed in six phases.

Ten age matched HS provided two separate speed-matched normative datasets for T0 and T1. For speed independent comparison for the PwMs *Z* scores were calculated for all their gait variables.

**Results:** In PwMS velocity increased significantly from T0 to T1 (0.74–0.90 m/s, *p* < 0.05). The activation profiles (API) of FPM and GCM of PwMS improved in pre-swing (*p* < 0.05): FPM (Mean [95% CI] [%]: T0: 12.5 [5.7–19.3] vs. T1: 9.0 [2.7–15.3]); GCM (T0: 26.7 [18.2–35.3] vs. T1: 24.5 [18.2–30.7]). This was associated with an increase in toe clearance (80.3 to 103.6 mm, *p* < 0.05) and a higher ankle power peak in pre-swing (1.53–1.93 W/kg, *p* < 0.05).

**Conclusion:** Increased gait speed of PwMS after intensive gait training was consistent with improvements in spatio-temporal gait parameters. The most important finding of this study was the re-organization of distal leg modules related to neurophysiological changes induced by rehabilitation. This was associated with an improved ankle performance.

## Introduction

Multiple sclerosis (MS) is a chronic inflammatory demyelinating disorder of the central nervous system (CNS) characterized by a progressive decline in various motor, sensory, and cognitive functions over the lifespan (1). Problems with mobility are evident in most persons with MS (PwMS), probably related to myelin damage which leads to adaptive changes in motor cortex and the spinal circuits (2, 3), as well as coordination problems, due to cerebellar circuit involvement (4). Resultant deficits in motor control and muscle weakness impact the gait function in PwMS and contribute to a reduction of participation in daily life activities (5, 6). Accordingly, regaining locomotor abilities is one of the primary goals of rehabilitation. Measuring the efficacy of rehabilitation on gait function at functional and neurophysiological levels is of utmost importance in this respect.

Clinical measures, frequently used to evaluate motor behavior and response to rehabilitation, are useful in describing the severity of mobility deficit, the functional walking status, and the amount of participation in daily life (5, 7). However, they do not inform on what may be the changes in neurophysiological or biomechanical mechanisms underlying an improvement in walking abilities following rehabilitation.

Spatiotemporal parameters and kinematic/kinetic variables derived through gait analysis have traditionally been used to objectively quantify walking abnormalities and biomechanical changes induced by rehabilitation (8). For example, it has been demonstrated (9–11) that PwMS walk slower and with a shorter stride length than healthy controls walking at matched speeds. Severini et al. (11) also found that PwMS showed decreased range of motion at hip, knee, and ankle while Filli et al. (10), in addition to the restrictions in joint excursion, found increased gait variability and asymmetry along with impaired dynamic stability. Such detailed information helps to describe the pathological gait patterns and to better understand the biomechanical contributions to possible recovery after rehabilitation but does not inform upon underlying changes in neuro-muscular control contributing to the improvement. Further, it would be important to verify whether observed post-treatment changes are related to a true physiological recovery rather than being a byproduct of the increased speed (10).

Modular organization of muscle coordination is thought to underlie motor control in both healthy individuals and individuals with neurological disorders (9, 12–14). Muscle synergies, derived from electromyographic signals during gait, are thought to reflect the underlying neural structures of muscle activation and local circuits (15, 16). Several studies have used muscle synergy analysis to model the complexity of motor control during gait, demonstrating that human gait can be described by a small set of robust synergies in healthy subjects (17–19) and in persons with neurological disorders (9, 20, 21).

Indeed, by using the muscle synergy approach as a framework, it was possible to study the neuromotor characteristics underlying walking in PwMS with moderate disability (9). The study of synergies in addition to gait biomechanics resulted in information not only on the biomechanical deficits present in gait of PwMS but also on corresponding deficits in neuromotor coordination. Main findings indicated that the walking deficits in PwMS were associated with muscle weakness and prolonged double support phases, corresponding to altered timing of the activation profiles of the distal motor modules (9). Importantly, PwMS had synergies number and module composition similar to healthy persons walking at the same speed, indicating a preserved organization of neuromotor control (9, 21).

Altogether, a combination of clinical scales, biomechanical, and muscle synergy analysis of gait allows understanding of deficits and limitations at the participation, activity, and impairment levels in persons with neurological disorders. This multivariate analysis can give information on functional performance as related to mobility, kinetics, kinematics, and neurological control of walking, all of which are essential for setting up effective customized treatments and for the evaluation of their efficacy. While muscle synergies and their relation to rehabilitation outcomes for persons post stroke (22) and with Parkinson's disorder (20) have been studied, to date no studies have yet been published that applied such a comprehensive approach in persons with MS.

In our recent work we described alterations in motor primitives of distal muscle synergies during walking of PwMS with respect to those of age and speed-matched healthy peers (9) in terms of important differences in timing activation across the gait cycle. In healthy individuals there are predominantly three to four modules that have been found to be related to walking (15, 23, 24). One or two modules are related to proximal muscle activity during gait. One usually consists mainly of activity from knee extensor, hip extensor, and abductor, which are primarily involved in early stance and late swing to prepare the leg for weight acceptance. The second proximal module consists mainly of hamstring activity from hip extensor and knee flexor, which are primarily involved in the early stance and late swing to extend the hip and decelerate leg swing (23). The two distal modules instead are related to propulsion and ground clearance and consist of the Forward Propulsion Module (FPM, mainly related to Soleus and Gastrocnemius) and Ground Clearance Module (GCM, mainly related to Tibialis Anterior and Rectus Femoris) (23). Using sEMG during overground gait, our prior investigation of module composition across PwMS and healthy controls revealed that when only three modules are identified, there was a merging of the two proximal modules (9). When timing of motor primitives (activity percentage indexes) was compared between the two groups, these proximal modules (both when merged and separate) resulted similar between PwMS and healthy individuals. On the other hand, timing of the motor primitives of the distal modules (FPM and GCM) results were altered in PwMS, regardless of module number. In addition, biomechanical gait deficits (i.e., reduced push-off and toe clearance in swing) were found to be consistent with impairments in activation and coordination of these distal synergies. This corroborated findings of others that have identified the reduction of (i) propulsion and (ii) foot-ground clearance during swing to be among the most disabling deficits in gait of PwMS (25, 26).

To exploit the clinical application of this information and use it to optimize/tailor rehabilitation interventions for PwMS, a crucial step is to evaluate if and how altered distal muscle synergies and their associated biomechanical parameters can be improved by rehabilitation.

Therefore, the focus of this article is on neuromotor recovery in distal parameters occurring with successful gait rehabilitation. More specifically, we investigated changes in neuromodular organization of FPM and GCM, and in propulsion and foot-ground clearance, of PwMS following a gait rehabilitation that lead to clinically meaningful increase in gait speed. These changes were investigated relative to healthy individuals walking at matched pre and post intervention speeds. We hypothesized that greater natural speed of walking would be associated with improvement in ankle control and associated biomechanical parameters in line with changes of healthy individuals. Further, we also investigated whether changes in the various gait parameters were proportional to speed changes in the PwMS.

## Methods

### Participants

Participants were 11 adults with relapsing-remitting or secondary progressive MS according to the 2005 McDonald criteria (27) who had volunteered for a larger controlled intervention trial carried out at IRCCS Don Gnocchi Foundation, Milan, Italy, in the period from October 2012 to April 2018 (see Table 1 for demographic information). From the entire dataset only the PwMS who agreed to undergo a complete gait analysis (electromyography, kinematic, and kinetics of the lower limb) pre- and post-intervention on treadmill (28) were considered for the present study. Of those, we analyzed gait data of 11 participants that had increased their gait speed by more than 15%. Inclusion criteria for PwMS were: diagnosis of multiple sclerosis according to the criteria of McDonald, Expanded Disability Status Scale (EDSS) ≤ 7 (29), age between 18 and 75 years, capacity to stand 30 s and walk 10 m with or without an assistive device (but without assistance from another person), capacity to understand and follow instructions, and stable neurological condition. Exclusion criteria: presence of any musculoskeletal and/or other neurologic pathologies that could influence gait and balance functions, presence of severe cardiovascular disorders.

**Table 1:** Individual demographic and clinical characteristics of PwMS and number of synergies at baseline and post-intervention.

**Subjects with MS**	**SEX**	**AGE [yrs]**	**ONSET [yrs]**	**EDSS**	**DGI**	**TUG [s]**	**2MWT [m]**	**N synergies (baseline)**	**N synergies (post)**
S1	F	61	13	6	12	13.5	84	3	4
S2	M	40	11	7	11	21	41	3	4
S3	F	54	25	6	13	11.3	128	3	3
S4	M	39	2	3	12	10.5	135	4	4
S5	M	36	13	4	22	8.3	155	4	4
S6	F	42	21	5	17	9.9	98	3	3
S7	F	68	19.5	5	17	15.1	75	4	3
S8	F	53	19	5	18	9.9	99	4	4
S9	M	64	23	4	14	15.9	122	3	4
S10	F	42	22	6	8	14.7	82	4	4
S11	F	54	25	6	9	14.1	113	4	3
Mean		50.3	17.6	5.2	13.9	13.1	102.9	3.5	3.6
Standard deviation		7.1	11.1	1.2	2	4.2	32.1	0.5	0.5

*PwMS, Persons with Multiple Sclerosis; MS, Multiple Sclerosis; EDSS, Expanded Disability Status Scale; DGI, Dynamic Gait Index; TUG, Timed Up and Go; 2MWT, Two Minute Walking Test; N, Number; F, female; M, male*.

Additionally, we analyzed gait data from 10 age-matched healthy controls (HS) (age Mean 43.1 SD 14.6 years, 6 Female and 4 Males) to derive biomechanical and muscle synergies reference data. Inclusion criteria for these healthy controls were: exhibiting normal joint range of motion and muscle strength, without any neuromuscular and balance deficits that could interfere with their gait.

The experimental protocol was approved by the institutional Ethics Committee (Comitato Etico Fondazione Don Carlo Gnocchi) and was carried out according to ethical standards of the Declaration of Helsinki. All participants signed an informed consent.

### Experimental Set Up and Procedures

We collected demographic and clinical variables according to study protocol [see Jonsdottir et al. (28)]. All participants with MS were evaluated with the following validated clinical scales: the 2 Minute Walking Test (2MWT) for gait endurance (30), the 10 meter timed walk (10MTW) for gait speed (31), and the Berg Balance scale for standing balance (32). The evaluation was carried out at baseline (T0) and after an intervention period (T1) by physical therapists blinded to the original study's group assignment (28).

Overground gait analysis of the PwMS participating in the present study and the HS was performed in a gait analysis lab. Kinematic, kinetic, and electromyography (EMG) data were thus collected both from healthy subjects (once) and from the participants affected by multiple sclerosis (twice, at T0 and T1). Kinematic data were collected using a 9-camera SMART-D motion capture system (BTS, Milano, Italy) sampling at 200 Hz, while a force plate (Kistler, Winterthur, Switzerland), with 960 Hz sampling frequency, provided ground reaction force (GRF).

We used an 8-channel EMG system (BTS, Milano, Italy) to record EMG data at 1,000 Hz from the following muscles: tibialis anterior (TA), soleus (SO), medial gastrocnemius (MG), lateral gastrocnemius (LG), vastus medialis (VM), rectus femoris (RF), semitendinosus (SE), gluteus medius (GM). Seniam recommendations for sEMG recording procedures were followed (33). The EMG sensors were placed on the most affected side for participants with MS [selected according to item 13 of the Berg Balance Scale, Standing unsupported one foot in front (32); the worse performance when right or left foot was in back during the pose] and on the dominant side (the leg that was used to kick a ball) for control subjects.

All participants (PwMS and HS) were asked to perform at least 5 gait trials barefoot at their natural self-selected speed, and the HS also performed trials at slower speeds (SW), to provide a reference dataset at gait speed matched with that of PwMS. In each trial only the central stride (the one on the platform) was analyzed. Since (i) PwMS significantly increased walking speed from T0 to T1 and (ii) there are speed-dependent effects on the timing patterns of muscle activity and on kinematic and kinetic parameters (34), we extracted two different speed-matched normative datasets across all trials at different speeds recorded from HS. This procedure provides two speed-matched datasets for comparison with trials of PwMS, one for baseline assessment and another dataset for post-treatment trials.

To create a speed matched dataset at baseline we included only trials from healthy subjects with normalized speed under the threshold of 62.2% BH/s. This threshold corresponded to 90% of the maximum normalized speed of PwMS at baseline. Analogously, for the post intervention database we included only trials of healthy subjects with normalized speed smaller than 66.0% BH/s corresponding to 90% of the maximum normalized speed of PwMS post-intervention. This is the same procedure for speed matching used in our previous study (9).

We adopted the total-body LAMB marker set, which includes 29 retro-reflective markers (12 mm diameter) positioned on the head, upper limbs, trunk, pelvis, and lower limbs (35).

### Intervention

The intervention and the associated clinical results are described in detail in Jonsdottir et al. (28). Briefly, the participants with MS had received supervised treadmill training, 4–5 sessions per week, 20 sessions in total, and were part of a subgroup that had undergone gait analysis at baseline and post intervention. Each treadmill walking session lasted 30 min and was aimed at improving participants' resistance, walking velocity, balance, and cognitive functions during locomotion. The intervention was mostly focused on maximizing the amount and intensity of walking activities, both aerobically and with dual task motor and cognitive activities, with no specific focus on normalizing gait kinematics.

### Gait Data Processing

After data acquisition, we low-pass filtered the markers' trajectories at a cut-off frequency of 6 Hz. We computed the anthropometric parameters of each subject from markers' positions recorded during the calibration trial according to the LAMB protocol (35), and used for estimation of internal joint centers. We also computed joint kinematics according to the LAMB protocol (35) and we used inverse dynamics to compute moments and powers at the ankle, knee, and hip joints of the selected leg with EMG probes. Each trial included the single gait cycle performed on the force plate. For each participant we computed the average value of selected parameters and the average pattern of kinematic/kinetic and EMG variables across trials.

### Kinematic and Kinetic Variables

We calculated the following gait parameters:

- gait speed (m/s): ratio between the linear distance traveled by the hip joint centers' midpoint during a stride and the stride duration;
- stride length (m): linear distance traveled by ankle joint center, estimated as the midpoint between lateral and medial malleolus, during a stride;
- cadence (steps/min): was calculated as 60/(0.5^*^stride duration);
- step width (m): lateral distance between the ankle joint center of the right and the left leg at respective foot's heel strike;
- DS1 (%): percentage of double support during loading response phase, calculated as the ratio between the time from heel contact of the supporting foot to contralateral foot-off and the stride time;
- DS2 (%): percentage of double support during pre swing phase, calculated as the ratio between the time from heel contact of the contralateral foot to supporting foot-off and the stride time;
- Maximal foot clearance (mm): maximal height of toe marker during the second half of swing phase, mainly related to the GCM;
- peak of ankle power (W/kg): maximal values of ankle power in stance phases, mainly related to FPM.

Heel contact and toe off were defined from the presence or absence of GRF data, respectively (36).

### EMG Processing

We high pass filtered the EMG signal with a cutoff frequency of 40 Hz, rectified and then low pass filtered with a cutoff frequency of 10 Hz, using a 4th order Butterworth filter. To focus on temporal dissimilarities in EMG, we normalized the signal of each muscle to its peak value across all recorded trials (14, 23). All data were time normalized to 100% of the gait cycle and subsequently averaged among trials of each subject.

We extracted muscle synergies using non-negative matrix factorization (NNMF) (37). For each participant the EMGs were combined into an *m* × *t* matrix, where *m* indicates the number of muscles and *t* is the time base (*t* = averaged stride ×101). We repeated the synergy extraction 50 times. The solution that accounted for >90% of the EMG variability for each muscle was selected, thus obtaining two matrices for each extracted muscle synergy: an *m* × *1* array, which specifies the relative weighting of each muscle in the module (module composition) and an *1* × *t* array, which specifies the activation timing profile of the module (9).

We calculated the following muscle synergies parameters:

- activation percentage index: for each gait phase [Early stance (P1), Mid stance (P2), Terminal stance (P3), Pre swing (P4), Early swing (P5), Late swing (P6)] each synergy's activation profile was integrated over 100% of the gait cycle and the percentage of such activation area was calculated within the specific phase;
- module similarity: Pearson's correlation coefficient of each module between each PwMS and the average module of the speed-matched control group. Higher correlations indicate more similarity in module compositions.

We calculated the activation percentage indexes for each participant, both for the PwMS and healthy subjects, while the module similarity parameter was calculated only in the multiple sclerosis group relative to the average of the speed matched control group.

We analyzed biomechanical and EMG measures with Matlab (The Mathworks Inc., Natick, MA).

### Statistical Analysis

We used *t*-tests for independent samples to compare age, body mass, and body weight between PwMS and HS, and a Chi-square test to compare gender (female/male) between groups (PwMS and HS).

At each time point (baseline or post-intervention), we used independent samples *t*-tests to compare gait and muscle synergies parameters between PwMS and speed-matched healthy subjects.

For comparison within the group of PwMS (pre- vs. post-intervention), we transformed the values of spatio-temporal and muscle synergy parameters for each PwMS to *z* scores (see Equation 1) resulting in parameter values independent of speed changes. This was done in order to analyze the deviation from the reference speed-matched data to account for the change in gait speed from baseline to post-intervention. P is the parameter, μ_speed−matchedHS_ and σ_speed−matchedHS_ are the mean value and the standard deviation, respectively, of that parameter for the reference control group at the matched speed.

(1) $$\begin{array}{r} Zscore_{P}=\frac{P-\mu_{speed-matched\;HS}}{\sigma_{speed-matched\;HS}} \end{array}$$

Subsequently, we ran paired *t*-tests on the *Z* scores to evaluate the pre- to post-intervention changes induced by the rehabilitation treatment. A significant change in *Z* scores indicated a change of that specific parameter from T0 to T1 beyond the changes due merely to a gait speed increase, and thus attributable to a response to treatment that went beyond speed dependent improvement. We summarized and tabulated the values of analyzed parameters as means and with 95% of the confidence interval. We considered *P* < 0.05 as statistically significant and 0.05 ≤ *P* < 0.1 as near-significant trends (38).

We performed all statistical analyses using Matlab (The Mathworks Inc., Natick, MA).

## Results

### General Demographics and Clinical Characteristics

Demographic characteristics of the participants and outcome of clinical scales are depicted in Table 1 individually and as a group mean. The general picture indicates a sample of patients with moderate mobility difficulties. We did not observe any differences in sex (*P* = 0.86), body mass, or height (Mean [95 CI%] [kg] PwMS vs. HS, 59.2 [52.8–65.5] vs. 65.8 [54.9–76.7] *P* = 0.24; [cm] 165.0 [159.1–171.0] vs. 168.1 [161.0–175.2] *P* = 0.46) between PwMS and HS.

### Spatio-Temporal Gait Parameters

All PwMS underwent gait analysis assessments autonomously without walking aids under the supervision of a physiotherapist.

See Table 2 for all gait variables for healthy controls and PwMS. Regarding the PwMS, average gait speed was 0.74 m/s (95% CI, 0.56, 0.92) at baseline and then increased to 0.90 m/s (0.71, 1.08) after the intervention, showing a statistically and clinically significant increase of 33.1% (*P* = 0.024). Gait speed of healthy subjects was matched at pre- and post-rehabilitation, resulting in a matched gait speed of 0.71 m/s (0.67, 0.76) at baseline and 0.93 m/s (0.84, 1.01) at post-intervention (*P* = 0.74 and *P* = 0.88, respectively, at baseline and post-intervention).

**Table 2:** Gait parameters and *Z-*scores of persons with Multiple Sclerosis (PwMS) at baseline (T0) and after treatment (T1).

		**HS Mean (95% CI)**		**PwMS Mean (95% CI)**		**PwMS** ***Z*****-score**	
		**Speed-Matched at T0**	**Speed-Matched at T1**	**T0**	**T1**	**T0**	**T1**
Gait Speed	[ms^−1^]	0.71 (0.67, 0.76)	0.93 (0.84, 1.01)^*^	0.74 (0.56, 0.92)	0.90 (0.71, 1.08)	0.35 (−2.40, 3.09)	−0.26 (−1.88, 1.37)^b^
Cadence	[steps min−1]	81.2 (71.1, 91.3)	93.2 (84.3, 102.0)^*^	88.8 (75.3, 102.4)	94.6 (80.7, 108.4)	0.54 (−0.42, 1.50)	0.11 (−1.01, 1.24)
Stride Length	[m]	1.10 (1.05, 1.16)	1.20 (1.14, 1.26)^*^	0.97 (0.83, 1.11)	1.09 (0.97, 1.22)^*^	−1.77 (−3.62, 0.08)	−1.24 (−2.66, 0.19)
Step Width	[mm]	88.2 (68.4, 107.9)	85.5 (70.1, 101.0)	149.4 (127.4, 171.5)^+^	147.5 (118.7, 176.3)^+^	2.22 (1.42, 3.02)	2.87 (1.54, 4.21)
Double Support early stance (DS1)	[%]	16.7 (15.4, 17.9)	13.9 (12.7, 15.2)	17.7 (13.4, 21.9)	15.3 (11.9, 18.7)	0.58 (−1.85, 3.02)	0.80 (−1.16, 2.75)
Double Support Pre Swing (DS2)	[%]	14.9 (13.5, 16.2)	13.2 (11.8, 14.6)	18.8 (12.9, 24.7)	15.9 (12.9, 18.9)	2.07 (−1.08, 5.21)	1.34 (−0.14, 2.83)
Peak Knee Flexion in swing	[deg]	60.3 (56.9, 63.6)	62.0 (59.2, 64.8)^*^	45.6 (32.4, 58.9)^+^	46.7 (32.6, 60.7)^+^	−3.12 (−5.95, −0.30)	−3.93 (−7.51, −0.35)
Foot Clearance	[mm]	123.6 (112.9, 134.4)	127.1 (118.0, 136.2)^*^	80.3 (56.2, 104.4)^+^	103.6 (81.7, 125.4)^*^	−2.89 (−4.50, −1.28)	−1.85 (−4.50, −1.28)^a^
Peak Ankle Plantarflexor Power	[W/Kg]	1.97 (1.58, 2.36)	2.55 (2.20, 2.91)^*^	1.53 (1.04, 2.03)	1.93 (1.50, 2.36)^*^^+^	−0.80 (−1.70, 0.11)	−1.26 (−2.13, −0.38)^b^

*For comparison, the values of the two speed-matched groups of healthy subjects (HS) are reported*.

+ *P < 0.05 at unpaired t-test between PwMS and HS before (pre) or after treatment (post) for the gait parameters*.

* *P < 0.05 at paired t-test within group before (pre) vs. after treatment (post) for the gait parameters*.

a *P < 0.05 at paired t-test within group before (pre) vs. after treatment (post) for the z-score of gait parameters*.

b *0.05 ≤ P < 0.1 at paired t-test within group before (pre) vs. after treatment (post) for the z-score of gait parameters*.

Stride length of PwMS was 0.97 m (0.83, 1.11) at baseline and post-intervention it was 1.09 (0.97, 1.22), showing a statistically significant increase of about 13% (*p* < 0.05). Cadence of PwMS was 88.8 ([steps/min], 95% CI 75.3–102.4) at baseline and 94.6 (80.7–108.4) following intervention. There was no change in step width or double support phases with increased speed neither in PWMS nor in HS. Step width in PwMS at baseline was 149.4 mm (Table 2, 95% CI 127.4, 171.5), much larger than in the HS (88.2 mm, 95% CI 68.4, 107.9). *Z* scores were not significant for any of the above parameters.

### Number of Modules and Module Composition

Among the healthy subjects (*N* = 10), 9 persons had 4 modules and one person had 3 modules when walking at the slower reference gait speed matching the pre-intervention gait speed of the PwMS, while at the higher reference gait speed, 6 persons (60%) had 4 modules and (40%) 4 persons had 3 modules (Table 1).

Of PwMS (*N* = 11), at baseline 6 persons (55%) had 4 modules and 5 persons (45%) had 3 modules. Post rehabilitation (Table 1) 7 PwMS had 4 modules (64%) and 4 persons had 3 modules (36%), indicating that the complexity of motor control was similar to that of healthy subjects at both reference speeds. There were no significant differences in demographic or clinical characteristics between subjects with 4 modules or 3 modules, except onset of MS where the persons with 3 synergies had an earlier onset of MS.

Module similarity of each module between each PwMS and the average module of the speed-matched control group was verified with the Pearson's correlation coefficient. At baseline module composition of the FPM in PwMS was similar to that of the HS, having a mean value >0.75 (Mean [95% CI], T0 0.79 [0.68–0.89]), while the overall composition of the GCM was less similar to that of the HS (T0 0.63 [0.43–0.83]). At T1 the module composition of muscle synergies of PwMS (Mean [95% CI] T1, FPM 0.85 [0.76–0.94]; GCM 0.76 [0.65–0.88]) increased in similarity to HS, although T0 to T1 difference on similarities were not statistically significant (*P* = 0.34 and *P* = 0.25, respectively, for FPM and GCM).

### Module Activation Indexes and Profiles

#### Forward Propulsive Module (FPM)

**Figure 1:**
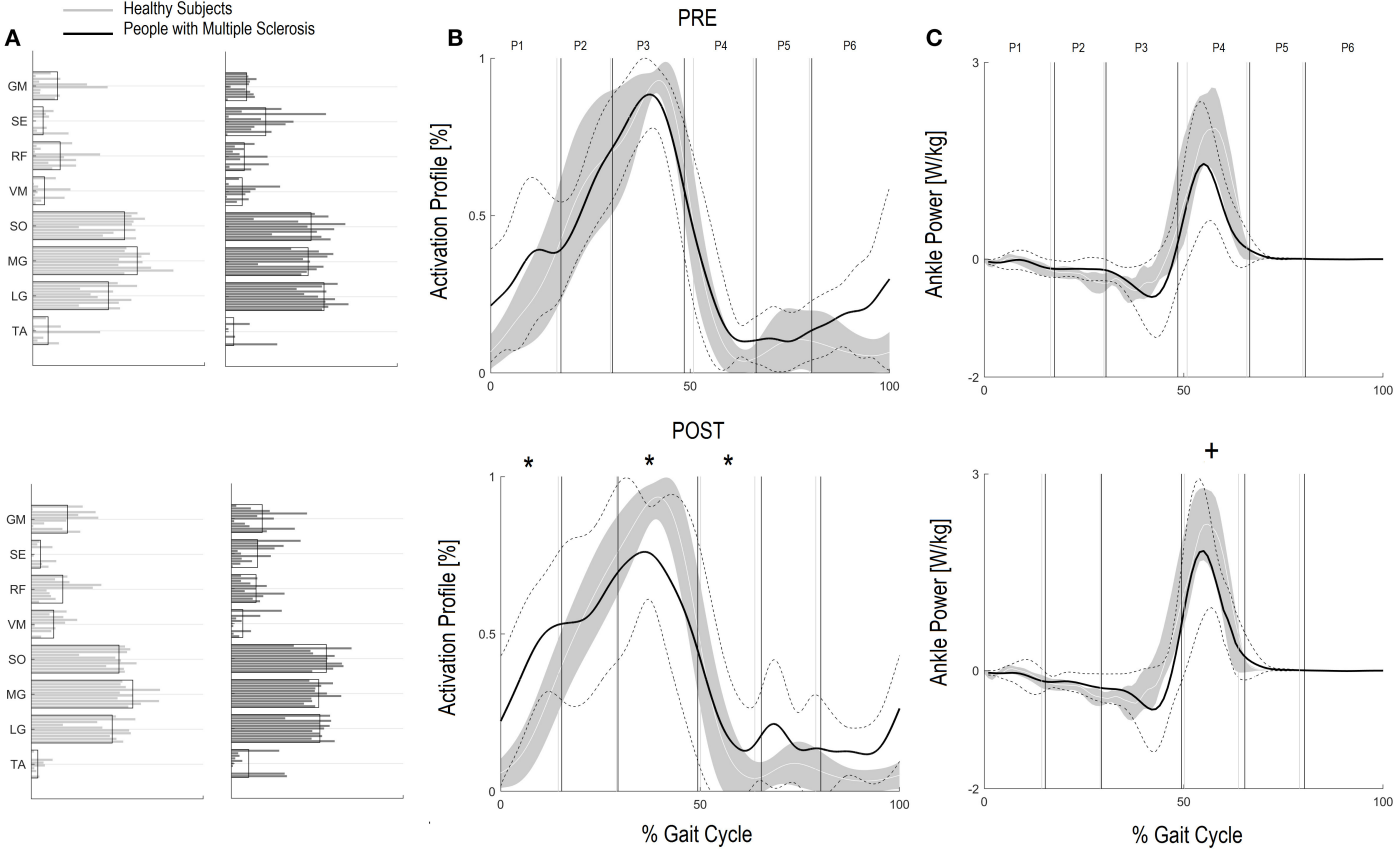
Forward Propulsion Muscle Synergies (FPM) and Kinetics of Ankle joint. Weightings **(A)** and activation profile **(B)** of FPM and ankle power curve **(C)** for persons with multiple sclerosis (PwMS) pre (top panel) and post (bottom panel) treatment and speed-matched healthy subjects (HS). In **(B,C)** The solid black line represents the averaged profile of PwMS and the dashed black lines represent ± SD of PwMS curves. Vertical lines (solid gray line—healthy subjects and solid black line—PwMS) indicate the six phases of a normalized gait cycle (P1, early stance; P2, mid stance; P3, terminal stance; P4, pre swing; P5, early swing; P6, late swing). Range of HS normality is reported in gray. *p < 0.05 at paired *t*-test of *Z*-score, pre- vs. post-treatment, for activation percentage index of FPM **(B)** or peak ankle power parameter **(C)** in PwMS. +0.05 < *p* < 0.1 at paired *t-*test of *Z*-score, pre- vs. post-treatment, for activation percentage index of FPM **(B)** or peak ankle power parameter **(C)** in PwMS.

Regarding the activation percentage index in FPM (Table 3, Figures 1A–C), at baseline PwMS was different from speed matched HS in early stance (P1) (HS vs. PwMS, 10.4 [8.0–12.8] vs. 15.6 [10.9–20.3], *p* = 0.05), mid stance (P2) (23.4 [19.5–27.4] vs. 18.1 [13.8–22.4] *p* = 0.06) terminal stance (P3) (51.8 [46.8–56.8] vs. 41.0 [33.8–48.2] *p* = 0.01) and late swing (P6) (3.9 [1.6–6.3] vs. 9.2 [6.3–12.2] *p* = 0.01), demonstrating difficulties in single and double support phases of gait. Regarding the comparison with HS, post intervention PwMS recovered mid stance where the activation index increased and become more similar to the physiological one (HS vs. PwMS 26.0 [22.1–29.8] vs. 22.7 [15.4–29.9], *P* = 0.39), while in the other gait phases the activation indexes remained different (*P* < 0.05) from HS. Looking at Figure 1B, it is noticed that in pre swing (P4) and early swing (P5) the morphology of activation profile of PwMS was more similar to the HS, recovering in early swing a second physiological activation peak as occurs in HS, even if anticipated in time.

**Table 3:** Activation percentage indexes (API) and *Z* scores of the Forward Propulsive Module activation profile during the gait phases for persons with Multiple Sclerosis (PwMS) at baseline (T0) and after treatment (T1).

**Parameters**		**HS Mean (95% CI)**		**PwMS Mean (95% CI)**		**PwMS** ***Z*****-score**	
		**Speed-Matched at T0**	**Speed-Matched at T1**	**T0**	**T1**	**T0**	**T1**
P1 Early Stance	[%]	10.4 (8.0, 12.8)	7.3 (5.4, 9.2)^*^	15.6 (10.9, 20.3)	16.7 (12.4, 21.1)^+^	1.57 (0.14, 3.00)	3.61 (1.94, 5.27)^a^
P2 Mid Stance	[%]	23.4 (19.5, 27.4)	26.0 (22.1, 29.8)	18.1 (13.8, 22.4)	22.7 (15.4, 29.9)	−0.96 (−1.72, −0.19)	−0.62 (−1.96, 0.72)
P3 Terminal Stance	[%]	51.8 (46.8, 56.8)	53.9 (50.3, 57.6)	41.0 (33.8, 48.2)^+^	38.2 (32.1, 44.4)^+^	−1.55 (−2.58, −0.52)	−3.06 (−4.26, −1.86)^a^
P4 Pre Swing	[%]	7.2 (4.4, 10.1)	7.0 (3.4, 10.5)	12.5 (5.7, 19.3)	9.0 (2.7, 15.3)^*^	1.32 (−0.37, 3.02)	0.40 (−0.87, 1.67)^a^
P5 Early Swing	[%]	3.2 (1.5, 4.9)	3.1 (1.6, 4.5)	3.6 (2.0, 5.2)	6.0 (2.5, 9.4)	0.16 (−0.49, 0.82)	1.40 (−0.28, 3.09)
P6 Late Swing	[%]	3.9 (1.6, 6.3)	2.8 (1.3, 4.3)	9.2 (6.3, 12.2)^+^	7.4 (4.7, 10.2)^+^	1.64 (0.74, 2.54)	2.22 (0.90, 3.53)

*The same indexes are reported for the two speed-matched datasets of healthy subjects (HS)*.

+ *P < 0.05 at unpaired t-test between PwMS and HS before (pre) or after treatment (post)*.

* *P < 0.05 at paired t-test within group before (pre) vs. after treatment (post)*.

a *P < 0.05 at paired t-test within group before (pre) vs. after treatment (post) for the z-score of gait parameters*.

On analysis of *z* scores of activation percentage indexes in PwMS (pre- vs. post-intervention) there was a statistically significant change in activation profiles following intervention in early stance where activation increased (worsening compared to HS), in terminal stance where there was a reduction of the activation (worsening), in pre swing (improvement) (Table 3, *P* = 0.01, *P* < 0.01 and *P* = 0.03, respectively), and a near significant increase in early swing (*P* = 0.10).

#### Kinetic Parameter Regarding the Forward Propulsive Module (FPM)

Corresponding to changes in the activation percentage indexes, the peak ankle power (Table 2), related to the push off deficit, increased in PwMS from 1.53 W/kg (1.04–2.03) at baseline to 1.93 W/kg (1.50–2.36) post-intervention, a 21% difference (*P* = 0.05). As a reference, HS presented values of 1.97 W/kg (1.58–2.36) and 2.55 W/kg (2.20–2.91) at the correspondent pre and post gait speeds (Table 2). Only at post-intervention the peak ankle power was different between HS and PwMS (*P* = 0.14 and *P* = 0.02, respectively, at pre and post intervention).

Within group observation of *Z* scores of PwMS revealed that there was a statistical trend for an increase in peak ankle power from baseline to post-intervention (Table 3 *P*-value of *Z*-score, *P* = 0.07) indicating an improvement in peak ankle power that went beyond the increase of speed. Since this is an important parameter to consider, we further investigated the changes within the individual PwMS. Of the 11 subjects, six PwMS improved from T0 to T1 by more than 15% of their baseline value, three PwMS improved between 10 and 15%, and two PwMS decreased their peak ankle power by 3 and 6%, respectively.

### Ground Clearance Module (GCM)

**Figure 2:**
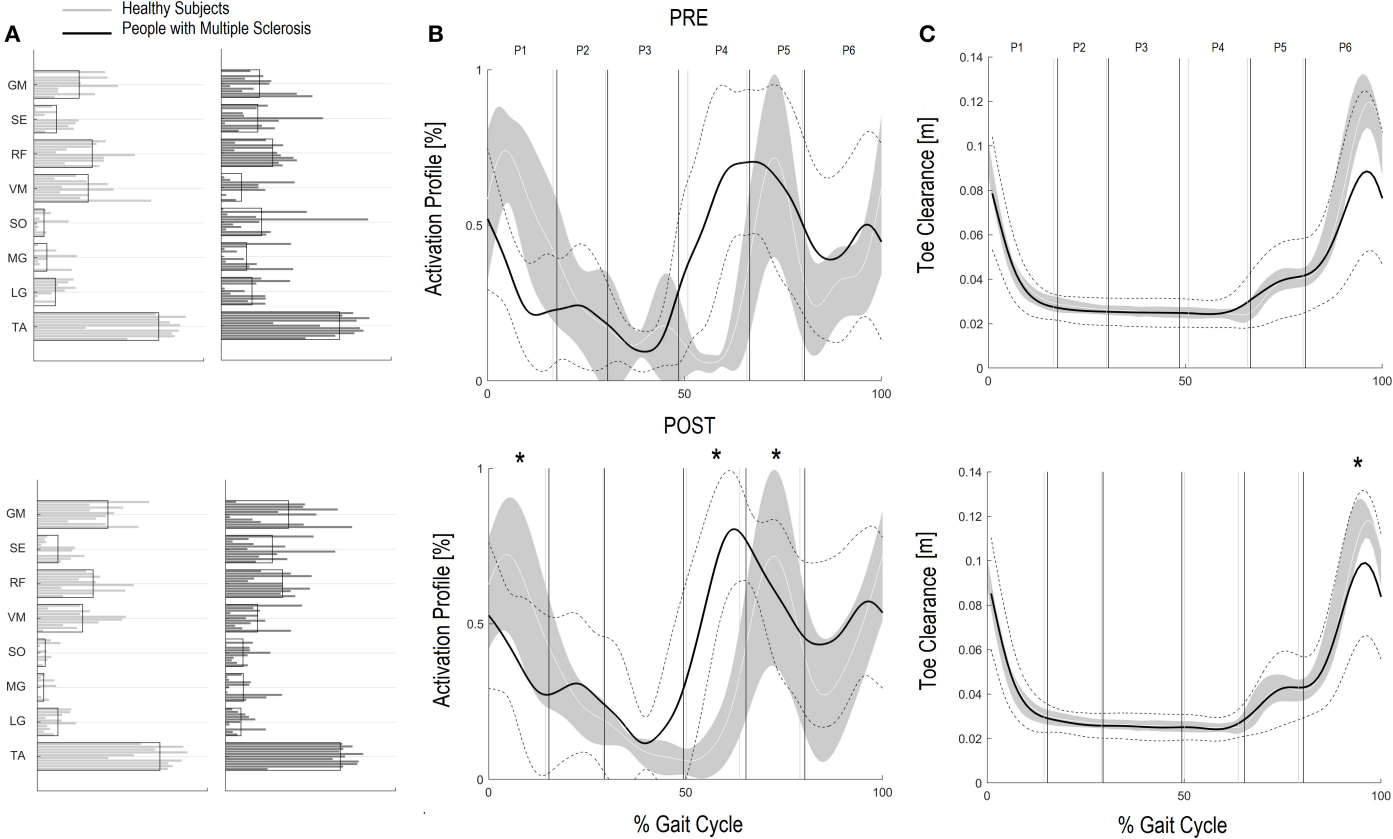
Ground Clearance Muscle Synergies (GCM) and Foot clearance. Weightings **(A)** and activation profile **(B)** of GCM and toe marker trajectory curve **(C)** for persons with multiple sclerosis (PwMS) pre (top panel) and post (bottom panel) treatment and speed-matched healthy subjects (HS). In **(B,C)** the solid black line represents the averaged profile of PwMS and the dashed black lines represent ± SD of PwMS curves. Vertical lines (solid gray line—healthy subjects and solid black line—PwMS) indicate the six phases of a normalized gait cycle (P1, early stance; P2, mid stance; P3, terminal stance; P4, pre swing; P5, early swing; P6, late swing). *p < 0.05 at paired *t*-test of *Z*-score, pre- vs. post-treatment, for activation percentage index of GCM **(B)** or toe clearance **(C)** in PwMS. +0.05 < *p* < 0.1 at paired *t*-test of *Z*-score, pre- vs. post-treatment, for activation percentage index of GCM **(B)** or toe clearance **(C)** in PwMS.

Regarding the GCM activation percentage index (Table 4 and Figures 2A–C) representing the synergy that controls clearance and loading response, at baseline the MS Group was different from speed matched HS in early stance (Table 4, HS vs. PwMS 26.4 [17.7–35.1] vs. 16.3 [9.8–22.9], *P* = 0.03), and pre swing (Table 4, 6.6 [2.9–10.2] vs. 26.7 [18.2–35.3], *P* < 0.01). The GCM, that in healthy subjects was mainly activated in early swing, in PwMS was activated in pre swing indicating an anticipated activation of tibialis anterior and rectus femoris muscles. With respect to the HS, at post intervention the MS Group recovered GCM activation index at early stance that became more similar to HS (HS vs. PwMS *P* = 0.15), while in pre swing (*P* < 0.01) and early swing (*P* = 0.04), it remained different from HS. Nonetheless, upon scrutiny of Figure 2B it is apparent that the activation profiles around pre swing and early swing (P4–P5) becomes sharper and more physiologically similar to that of the HS.

**Table 4:** Activation percentage index (API) and *Z* scores of the Ground Clearance Module activation profile during the gait phases for persons with Multiple Sclerosis (PwMS) at baseline (T0) and after treatment (T1).

**Parameters**		**HS Mean (95% CI)**		**PwMS Mean (95% CI)**		**PwMS** ***Z*****-score**	
		**Speed-Matched at T0**	**Speed-Matched at T1**	**T0**	**T1**	**T0**	**T1**
P1 Early Stance	[%]	26.4 (17.7–35.1)	20.8 (12.1–29.6)^*^	16.3 (9.8–22.9)^+^	14.3 (9.6–19.0)	−0.90 (−1.40, −0.41)	−0.53 (−0.92, −0.15)^a^
P2 Mid Stance	[%]	10.3 (6.2–14.4)	11.2 (7.7–14.8)	8.0 (4.0–11.9)	9.2 (5.9–12.5)	−0.67 (1.12, −0.22)	−0.41 (−1.07, 0.26)
P3 Terminal Stance	[%]	8.4 (6.0–10.8)	7.3 (5.1–9.4)	6.4 (4.9–8.0)	7.2 (4.0–10.4)	−0.26 (−0.68, 0.16)	−0.01 (−1.06, 1.04)
P4 Pre Swing	[%]	6.6 (2.9–10.2)	6.2 (3.7–8.7)	26.7 (18.2–35.3)^+^	24.5 (18.2–30.7)^+^	10.66 (7.17, 14.15)	5.18 (3.41, 6.96)^a^
P5 Early Swing	[%]	25.3 (18.6–32.1)	30.3 (23.0–37.5)^*^	23.0 (17.2–28.8)	22.4 (18.2–26.7)^+^	−0.32 (−0.94, 0.30)	−0.77 (−1.19, −0.36)^a^
P6 Late Swing	[%]	23.0 (16.5–29.5)	24.2 (19.5–28.8)	19.5 (13.1–25.9)	22.3 (18.1–26.6)	−0.37 (−1.07, 0.32)	−0.29 (−0.94, 0.37)

*The same indexes are reported for the two speed-matched groups of healthy subjects (HS)*.

+ *P < 0.05 at unpaired t-test between PwMS and HS before (pre) or after treatment (post)*.

* *P < 0.05 at paired t-test within group before (pre) vs. after treatment (post)*.

a *P < 0.05 at paired t-test within group before (pre) vs. after treatment (post) for the z-score of gait parameters*.

Regarding *Z* scores of PwMS, post-intervention there were significant changes in activation indexes in early stance (improvement), pre swing (improvement), and early swing (worsening) (*P* = 0.01, *P* < 0.01, and *P* = 0.03, respectively) with respect to baseline.

#### Kinematic Parameter Regarding the Ground Clearance Module (GCM)

Absolute foot-ground clearance (Table 2), defined as maximal height of toe marker during the second half of swing phase, increased with increased speed in both groups (T0–T1): in PwMS from 80.3 mm (95% CI 56.2–104.4) to 103.6 mm (81.7–125.4) (*P* < 0.01); and in HS from 123.6 mm (112.9–134.4) to 127.1 mm (118.0–136.2) (*P* = 0.03).

The *z*-score of absolute foot clearance in PwMS increased significantly from baseline to T1 (*P* < 0.01).

## Discussion

Objectives of this study were (i) to investigate the effect of rehabilitation induced improvement in preferred gait velocity on (1) leg distal modules composition and their timing activation profile, and (ii) on walking performance in PwMS with emphasis on gait parameters related to forward propulsion and ground clearance.

Resultant changes in spatiotemporal parameters were mostly proportionate to changes in speed, indicating the function was recovered through improvement in physiological strategies. Importantly, there were indications that distal muscle synergies and biomechanical parameters, e.g., peak ankle power and ground clearance, may be influenced by rehabilitation. In fact, when effects of training on peak ankle power and ground clearance were corrected for confounding by increases in walking speed, there was a response to rehabilitation that went beyond that being proportional to change in speed. Taken together these results indicate an improvement in distal neuromotor locomotion function and a recovery in response to treatment.

The PwMS in the present study were quite heterogeneous with moderate to severe gait disability. Baseline gait analysis confirmed findings from previous studies on gait performance in PwMS demonstrating reduced stride length, increased cadence and increased step width, with reduction of propulsive work at the ankle, decreased knee flexion during swing, and decreased foot clearance compared to speed matched healthy subjects (9, 39, 40). They did, however, all respond well to the intensive training on treadmill with an average increase in gait speed of 33% from baseline to post intervention. The change in gait speed was important at the participation level in that the group went from having a gait speed descriptive of a limited community ambulation to being in the unlimited category of community ambulation post intervention (>0.80 m/s) (41). These changes were corroborated with results from the gait analysis. Most spatiotemporal gait parameters improved in line with speed related changes in healthy subjects indicating PwMS can increase self-selected velocity without using compensatory strategies. Step width, instead, was a parameter independent from gait speed in both groups and remained much larger in PwMS also following intervention, consistent with known dynamic balance insecurities in that population (42, 43).

Regarding motor synergies, analysis of baseline neurophysiological parameters confirmed that PwMS tend to have a preserved number of modules in the lower leg during gait (three or four modules), and preserved modular composition within the distal leg modules related to propulsion and ground clearance (9, 21). On the contrary, timing of the modular muscle activations (motor primitives) differed from those of healthy controls walking at comparable speeds. Investigation of motor module number at baseline confirmed results from our previous work on PwMS (9) that demonstrated that PwMS recruit either 3 or 4 motor modules at preferred gait speed reflecting the preserved complexity of motor control (13). This is consistent with findings for persons with Parkinson's disorder that tend to have a preserved motor module number during gait (20), while on the contrary findings from persons post-stroke (22), persons with spinal injuries (44), and cerebral palsy (45) suggest an influence of the disorder on the motor module number during gait. This may be due to different central nervous system damages, since multiple sclerosis typically causes diffuse central and neuronal damage and atrophy that might allow preservation of neural control signals and synergy complexity (46).

### Distal Modules and Gait Biomechanics

Efficacious gait rehabilitation (augmented preferred gait speed) led to a consolidation of the muscle weightings and improved consistency of the distal muscle synergies patterns, primarily with changes in motor primitives of the two distal modules. These changes were consistent with positive rehabilitation benefits in propulsion and absolute foot clearance, regardless of the change in speed.

Peak ankle power, the main kinetic parameter of the FPM (forward propulsive model) increased from baseline to post intervention with a positive trend of the treatment (Table 2, *P*-value of *Z*-score = 0.07). This is an important finding since it has been suggested that the ankle plantarflexion is the controlled variable in the gait cycle (47, 48). The intervention was quite intense, including both fast walking for aerobic training and dual motor tasks carried out with the treadmill in movement, that included walking on toes, doing long strides, high knee raises, etc. [see (28) for further details of the training protocol]. It is likely that these aspects of training facilitated an improved ankle plantarflexor neuromotor organization and peak ankle power during push off. Davies et al. (48) saw a similar effect on ankle plantarflexor control of PwMS after a neurorehabilitation protocol focusing on distal leg parameters with all of the outcome variables matching or trending toward those seen in healthy controls.

The results are also consistent with findings of Routson et al. (22) that saw treadmill training leading to better walking performance and modular organization in persons post stroke. An important finding of that study was that gait recovery was associated with improved motor primitives (activation profiles) in the distal modules, in particular in the FPM, likely contributing to improvement in biomechanical measures.

The suggestion is that gait specific training protocols have the potential to promote clinically relevant improvement in the ankle plantarflexor control as well as peak ankle power with beneficial effects on mobility parameters.

An increase in peak ankle power and improved distal modular activation are important for walking function in PwMS since plantarflexor weakness and ankle motor control are major deficits leading to difficulty in locomotion (9, 49). Improved ankle joint coordination during push off leads to more energy efficient gait, increased gait speed, and possibly easier participation in daily life community activities.

Additionally, since many falls in PwMS can be traced to tripping (50), the improved foot clearance points to safer locomotion and a potentially diminished risk of falling. Foot clearance occurs through coordinated movements of the leg joints and, in particular, with (i) a correct positioning of the foot and muscles recruitment in the terminal support phase, in order to prepare the leg for the swing phase and (ii) a coordinated recruitment of dorsiflexors during the swing phase (51). The foot clearance in the enrolled PwMS in this study was quite deficient. Their reduction in clearance during swing influences ankle joint position at the beginning of the stride, requiring greater ankle joint muscular coactivation (52). After the rehabilitation intervention foot clearance increased significantly by approximately 30% nearing values of healthy controls at matched speeds. In the PwMS it was the single most changed parameter when speed was accounted for (*Z* scores *P* < 0.01) indicating a strong therapeutic effect of the treatment. Upon scrutiny of the baseline GCM activation profile (see Figure 2B) it is evident that the modular impulse in healthy subjects occurs in the early swing phase of the gait cycle while in the PwMS the maximum value occurred earlier, in the pre swing phase, typical of inappropriate distal limb activation in persons with neurological disorders (53–55). This probably occurs due to the fact that, since the PwMS do not develop enough ankle power, they have to activate the dorsiflexors in early midstance and during late push off, preparing for lift off and elevation of foot during swing to reduce risk of tripping. Following the gait rehabilitation it was evident that they recovered a more physiological activation impulse in the GCM and had activation profiles that were more consistent with strategies observed in the HS (Figure 2B).

It has been suggested by many authors that the ankle plantarflexion is the controlled variable in the gait cycle, determining changes in speed (47, 48). In a recent work by Davies et al. (25) it was found that errors in the ankle plantarflexor force production were related to gait deficits of PwMS. They further suggested that the improvements seen in gait of PwMS following gait rehabilitation interventions were related to changes in the motor control of the ankle musculature. This is supported by our findings where gait rehabilitation resulted in improvement of dynamic motor control as evidenced by more appropriate activation profiles in FPM and GCM.

### General Considerations

High intensity mobility training provided to persons with moderate to severe disability from multiple sclerosis resulted in substantial increase in gait velocity. The overall improvements seen, including the increase in preferred speed, are mainly due to the benefits produced by the rehabilitation in domains related to muscle coordination and walking performance. The methodologies adopted for data analyses in this study [i.e., (i) the choice to create two databases of healthy subjects matched with speed of PwMS, respectively, at pre- and at post-intervention, and (ii) consequently the statistical analysis on the *Z*-score values] allowed the evaluation of the underlying neurophysiological recovery following rehabilitation. The results highlighted that the two main gait parameters related to distal muscle synergies control, the foot clearance and the peak ankle power, improved with reorganization at the modular level in PwMS. This was an effect of the treatment that went beyond preferred speed appropriate changes.

Those changes in preferred gait velocity were concurrent with improvements in distal neuromuscular and biomechanical aspects reflecting a recovery of motor control. Further investigation of modular organization in distal parameters demonstrated improved activation timing of plantarflexors in the push off and of dorsiflexors in mid/late stance corresponding to improved foot clearance. This was further confirmed by improvements in biomechanical walking parameters related to the distal modules indicating a potential reorganization at central and peripheral level. Modular reorganization in this context can be taken as a sign of neuroplasticity, a form of recovery in response to rehabilitation. To promote functional recovery and in order to stimulate neural plasticity and change modular organization, something (some activity or exercise) has to be done intensely, voluntarily, and for some time either by the person alone (starts exercising) or in collaboration with a therapist. Treadmill training might be a particularly appropriate approach to gait rehabilitation since it allows the PwMS to walk further and longer than might otherwise be possible. In this context it has been hypothesized that learning-dependent changes in Central Pattern Generator circuits occur primarily through rhythmic perceptual influences imposed by exercises, an example of which could be walking on treadmill during dual task activities [see gait protocol (28)]. The present analysis of distal leg muscle synergies during overground gait revealed an overall improvement in activation percentage indexes and correlated kinetic and kinematic parameters. In spite of this improvement altered early activation impulses were still evident in key activities of the gait cycle, in line with findings of Janshen et al. (21). Further research might consider adding an electrical stimulation or a biofeedback from plantarflexor muscles during gait activities on the treadmill to enhance even more timing of module activation. In this context, muscle synergies analysis as an adjunct to clinical and biomechanical analysis may become a useful tool for setting up increasingly efficacious rehabilitation protocols, and for evaluating and understanding changes due to rehabilitation at all levels of mobility.

Motor modules probably arise from neural plasticity in supraspinal and spinal structures and are shaped by regularities in biomechanical interactions with the environment (15). Effect of rehabilitation is interesting in this perspective. Task specific training should be intensive enough to harness use dependent neuroplasticity (56). The use of motor module analysis can help to highlight disease characteristic neuromotor deficits, as well as individuate individual specific neuromotor deficits. Further, the current results from PwMS indicate that motor module analysis can help in elucidating the effect of a therapeutic intervention. Most importantly, it can provide a framework for developing more efficacious therapies that enhance neural plasticity and promote motor recovery, both from a global perspective and an individual perspective.

### Limitations

There are limitations to this exploratory study. The number of enrolled subjects is low given the heterogeneity of the subjects and so the results of the study can only be generalized to a PwMS with similar moderate mobility disabilities. In this study we analyzed data only from PwMS that had augmented their gait velocity after rehabilitation. This means we cannot know if the participants that do not respond to rehabilitation by increasing gait velocity adapt their biomechanical and neurophysiological gait parameters. A future study with a larger number of subjects should be planned to confirm the findings, including also PwMS that do not respond to the rehabilitation, in a comparison analysis.

Other limitations include the methodology; muscle synergy extraction depends on methodological factors that have to be considered, however, our synergy analyses are comparable to those of studies already published that have investigated changes in response to rehabilitation in stroke patients (22) similar to that of our population with MS.

## Data Availability Statement

The datasets generated for this study are available on request to the corresponding author.

## Ethics Statement

The studies involving human participants were reviewed and approved by Fondazione Don Carlo Gnocchi. The patients/participants provided their written informed consent to participate in this study.

## Author Contributions

JJ, TL, MF, and DC: substantial contributions to the conception or design of the work or interpretation of data for the work. DA, EG, and AC: recruitment and treatment of participants and data acquisition. TL and IC: statistical analysis of the data. JJ, TL, and MF: drafting the work or revising it critically for important intellectual content. JJ, TL, MF, IC, DC, AC, DA, EG, and MR: final approval of the version to be published. TL, JJ, MF, DC, and MR: agreement to be accountable for all aspects of the work in ensuring that questions related to the accuracy or integrity of any part of the work are appropriately investigated and resolved. All authors: contributed to the article and approved the submitted version.

## Conflict of Interest

The authors declare that the research was conducted in the absence of any commercial or financial relationships that could be construed as a potential conflict of interest.

## References

[1] Popescu BFG Lucchinetti CF. Meningeal and cortical grey matter pathology in multiple sclerosis. BMC Neurol. (2012) 12:11. 10.1186/1471-2377-12-11 22397318 PMC3315403

[2] Compston A Coles A. Multiple sclerosis. Lancet. (2008) 372:1502–17. 10.1016/S0140-6736(08)61620-7 18970977

[3] Reich DS Lucchinetti CF Calabresi PA. Multiple sclerosis. N Engl J Med. (2018) 378:169–80. 10.1056/NEJMra1401483 29320652 PMC6942519

[4] Wilkins A. Cerebellar dysfunction in multiple sclerosis. Front Neurol. (2017) 8:312 10.3389/fneur.2017.00312 28701995 PMC5487391

[5] Cattaneo D Lamers I Bertoni R Feys P Jonsdottir J. Participation restriction in people with multiple sclerosis: prevalence and correlations with cognitive, walking, balance, and upper limb impairments. Arch Phys Med Rehabil. (2017) 98:1308–15. 10.1016/j.apmr.2017.02.015 28336344

[6] Carpinella I Gervasoni E Anastasi D Lencioni T Cattaneo D Ferrarin M. Instrumental assessment of stair ascent in people with multiple sclerosis, stroke, and parkinson's disease: a wearable-sensor-based approach. IEEE Trans Neural Syst Rehabil Eng Publ IEEE Eng Med Biol Soc. (2018) 26:2324–32. 10.1109/TNSRE.2018.2881324 30442611

[7] Bertoni R Jonsdottir J Feys P Lamers I Cattaneo D. Modified functional walking categories and participation in people with multiple sclerosis. Mult Scler Relat Disord. (2018) 26:11–8. 10.1016/j.msard.2018.08.031 30212768

[8] Jonsdottir J Recalcati M Rabuffetti M Casiraghi A Boccardi S Ferrarin M. Functional resources to increase gait speed in people with stroke: strategies adopted compared to healthy controls. Gait Posture. (2009) 29:355–9. 10.1016/j.gaitpost.2009.01.008 19211250

[9] Lencioni T Jonsdottir J Cattaneo D Crippa A Gervasoni E Rovaris M. Are modular activations altered in lower limb muscles of persons with multiple sclerosis during walking? Evidence from muscle synergies and biomechanical analysis. Front Hum Neurosci. (2016) 10:620. 10.3389/fnhum.2016.00620 28018193 PMC5145858

[10] Filli L Sutter T Easthope CS Killeen T Meyer C Reuter K. Profiling walking dysfunction in multiple sclerosis: characterisation, classification and progression over time. Sci Rep. (2018) 8:4984. 10.1038/s41598-018-22676-0 29563533 PMC5862880

[11] Severini G Manca M Ferraresi G Caniatti LM Cosma M Baldasso F. Evaluation of clinical gait analysis parameters in patients affected by multiple sclerosis: analysis of kinematics. Clin Biomech. (2017) 45:1–8. 10.1016/j.clinbiomech.2017.04.001 28390935

[12] Scano A Dardari L Molteni F Giberti H Tosatti LM d'Avella A. A comprehensive spatial mapping of muscle synergies in highly variable upper-limb movements of healthy subjects. Front Physiol. (2019) 10:1231. 10.3389/fphys.2019.01231 31611812 PMC6777095

[13] Allen JL Kesar TM Ting LH. Motor module generalization across balance and walking is reduced after stroke. J Neurophysiol. (2019) 122:277–89. 10.1152/jn.00561.2018 31066611 PMC6689783

[14] Routson RL Kautz SA Neptune RR. Modular organization across changing task demands in healthy and poststroke gait. Physiol Rep. (2014) 2:e12055–e55. 10.14814/phy2.12055 24963035 PMC4208640

[15] Ting LH Chiel HJ Trumbower RD Allen JL McKay JL Hackney ME. Neuromechanical principles underlying movement modularity and their implications for rehabilitation. Neuron. (2015) 86:38–54. 10.1016/j.neuron.2015.02.042 25856485 PMC4392340

[16] Safavynia S Torres-Oviedo G Ting L. Muscle synergies: implications for clinical evaluation and rehabilitation of movement. Top Spinal Cord Inj Rehabil. (2011) 17:16–24. 10.1310/sci1701-16 21796239 PMC3143193

[17] Ivanenko YP Poppele RE Lacquaniti F. Five basic muscle activation patterns account for muscle activity during human locomotion. J Physiol. (2004) 556:267–82. 10.1113/jphysiol.2003.057174 14724214 PMC1664897

[18] Monaco V Ghionzoli A Micera S. Age-related modifications of muscle synergies and spinal cord activity during locomotion. J Neurophysiol. (2010) 104:2092–102. 10.1152/jn.00525.2009 20685924

[19] Chvatal SA Ting LH. Common muscle synergies for balance and walking. Front Comput Neurosci. (2013) 7:48. 10.3389/fncom.2013.00048 23653605 PMC3641709

[20] Allen JL McKay JL Sawers A Hackney ME Ting LH. Increased neuromuscular consistency in gait and balance after partnered, dance-based rehabilitation in parkinson's disease. J Neurophysiol. (2017) 118:363–73. 10.1152/jn.00813.2016 28381488 PMC5501921

[21] Janshen L Santuz A Ekizos A Arampatzis A. Fuzziness of muscle synergies in patients with multiple sclerosis indicates increased robustness of motor control during walking. Sci Rep. (2020) 10:7249. 10.1038/s41598-020-63788-w 32350313 PMC7190675

[22] Routson RL Clark DJ Bowden MG Kautz SA Neptune RR. The influence of locomotor rehabilitation on module quality and post-stroke hemiparetic walking performance. Gait Posture. (2013) 38:511–7. 10.1016/j.gaitpost.2013.01.020 23489952 PMC3687005

[23] Clark DJ Ting LH Zajac FE Neptune RR Kautz SA. Merging of healthy motor modules predicts reduced locomotor performance and muscle coordination complexity post-stroke. J Neurophysiol. (2010) 103:844–57. 10.1152/jn.00825.2009 20007501 PMC2822696

[24] Singh RE Iqbal K White G Hutchinson TE. A systematic review on muscle synergies: from building blocks of motor behavior to a neurorehabilitation tool. Appl bionics Biomech. (2018) 2018:3615368. 10.1155/2018/3615368 29849756 PMC5937559

[25] Davies BL Hoffman RM Healey K Zabad R Kurz MJ. Errors in the ankle plantarflexor force production are related to the gait deficits of individuals with multiple sclerosis. Hum Mov Sci. (2017) 51:91–98. 10.1016/j.humov.2016.11.008 27923175

[26] Artoni F Fanciullacci C Bertolucci F Panarese A Makeig S Micera S. Unidirectional brain to muscle connectivity reveals motor cortex control of leg muscles during stereotyped walking. Neuroimage. (2017) 159:403–16. 10.1016/j.neuroimage.2017.07.013 28782683 PMC6698582

[27] Polman CH Reingold SC Edan G Filippi M Hartung HP Kappos L. Diagnostic criteria for multiple sclerosis: 2005 revisions to the “McDonald criteria.” Ann Neurol. (2005) 58:840–6. 10.1002/ana.20703 16283615

[28] Jonsdottir J Gervasoni E Bowman T Bertoni R Tavazzi E Rovaris M. Intensive multimodal training to improve gait resistance, mobility, balance and cognitive function in persons with multiple sclerosis: a pilot randomized controlled trial. Front Neurol. (2018) 9:800. 10.3389/fneur.2018.00800 30323787 PMC6172314

[29] Kurtzke JF. Rating neurologic impairment in multiple sclerosis: an expanded disability status scale (EDSS). Neurology. (1983) 33:1444–52. 10.1212/WNL.33.11.1444 6685237

[30] Gijbels D Eijnde BO Feys P. Comparison of the 2- and 6-minute walk test in multiple sclerosis. Mult Scler J. (2011) 17:1269–72. 10.1177/1352458511408475 21642370

[31] Kempen JCE de Groot V Knol DL Polman CH Lankhorst GJ Beckerman H. Community walking can be assessed using a 10-metre timed walk test. Mult Scler. (2011) 17:980–90. 10.1177/1352458511403641 21622593

[32] Berg K Wood-Dauphinee S Williams JI Gayton D Measuring balance in the elderly: preliminary development of an instrument. Physiother Canada. (1989) 41:304–11. 10.3138/ptc.41.6.304

[33] Hermens HJ Freriks B Disselhorst-Klug C Rau G. Development of recommendations for SEMG sensors and sensor placement procedures. J Electromyogr Kinesiol. (2000) 10:361–74. 10.1016/S1050-6411(00)00027-4 11018445

[34] Lencioni T Carpinella I Rabuffetti M Marzegan A Ferrarin M. Human kinematic, kinetic and EMG data during different walking and stair ascending and descending tasks. Sci Data. (2019) 6:309. 10.1038/s41597-019-0323-z 31811148 PMC6897988

[35] Rabuffetti M Marzegan A Crippa A Carpinella I Lencioni T Castagna A. The LAMB gait analysis protocol: definition and experimental assessment of operator-related variability. Proc Inst Mech Eng Part H J Eng Med. (2019) 233:342–53. 10.1177/0954411919827033 30706762

[36] Mickelborough J Van Der Linden ML Richards J Ennos AR. Validity and reliability of a kinematic protocol for determining foot contact events. Gait Posture. (2000) 11:32–7. 10.1016/S0966-6362(99)00050-8 10664483

[37] Lee DD Seung HS. Learning the parts of objects by non-negative matrix factorization. Nature. (1999) 401:788–91. 10.1038/44565 10548103

[38] Gelman A. P values and statistical practice. Epidemiology. (2013) 24:69–72. 10.1097/EDE.0b013e31827886f7 23232612

[39] Kasser SL Jacobs JV Foley JT Cardinal BJ Maddalozzo GF. A prospective evaluation of balance, gait, and strength to predict falling in women with multiple sclerosis. Arch Phys Med Rehabil. (2011) 92:1840–6. 10.1016/j.apmr.2011.06.004 21840497

[40] Benedetti MG Piperno R Simoncini L Bonato P Tonini A Giannini' S. Gait abnormalities in minimally impaired multiple sclerosis patients. Mult Scler. (1999) 5:363–8. 10.1177/135245859900500510 10516781

[41] Perry J Garrett M Gronley JK Mulroy SJ. Classification of walking handicap in the stroke population. Stroke. (1995) 26:982–9. 10.1161/01.STR.26.6.982 7762050

[42] Peebles AT Bruetsch AP Lynch SG Huisinga JM. Dynamic balance in persons with multiple sclerosis who have a falls history is altered compared to non-fallers and to healthy controls. J Biomech. (2017) 63:158–63. 10.1016/j.jbiomech.2017.08.023 28889946

[43] Lencioni T Anastasi D Carpinella I Castagna A Crippa A Gervasoni E Dynamic balance during level walking in patients affected by multiple sclerosis, stroke and parkinson's disease. Gait Posture. (2018) 66:S23–S4. 10.1016/j.gaitpost.2018.07.136

[44] Hayes HB Chvatal SA French MA Ting LH Trumbower RD. Neuromuscular constraints on muscle coordination during overground walking in persons with chronic incomplete spinal cord injury. Clin Neurophysiol. (2014) 125:2024–35. 10.1016/j.clinph.2014.02.001 24618214 PMC4133333

[45] Steele KM Rozumalski A Schwartz MH. Muscle synergies and complexity of neuromuscular control during gait in cerebral palsy. Dev Med Child Neurol. (2015) 57:1176–82. 10.1111/dmcn.12826 26084733 PMC4683117

[46] Schlaeger R Papinutto N Panara V Bevan C Lobach IV Bucci M. Spinal cord gray matter atrophy correlates with multiple sclerosis disability. Ann Neurol. (2014) 76:568–80. 10.1002/ana.24241 25087920 PMC5316412

[47] Honeine J-L Schieppati M Gagey O Do M-C. By counteracting gravity, triceps surae sets both kinematics and kinetics of gait. Physiol Rep. (2014) 2:e00229. 10.1002/phy2.229 24744898 PMC3966244

[48] Davies BL Arpin DJ Volkman KG Corr B Reelfs H Harbourne RT. Neurorehabilitation strategies focusing on ankle control improve mobility and posture in persons with multiple sclerosis. J Neurol Phys Ther. (2015) 39:225–32. 10.1097/NPT.0000000000000100 26247511

[49] Wagner JM Kremer TR Van Dillen LR Naismith RT. Plantarflexor weakness negatively impacts walking in persons with multiple sclerosis more than plantarflexor spasticity. Arch Phys Med Rehabil. (2014) 95:1358–65. 10.1016/j.apmr.2014.01.030 24582617 PMC4152915

[50] Carling A Forsberg A Nilsagård Y. Falls in people with multiple sclerosis: experiences of 115 fall situations. Clin Rehabil. (2018) 32:526–35. 10.1177/0269215517730597 28901164 PMC5865469

[51] Little VL McGuirk TE Patten C. Impaired limb shortening following stroke: what's in a name? PLoS ONE. (2014) 9:e110140. 10.1371/journal.pone.0110140 25329317 PMC4199676

[52] Lencioni T Piscosquito G Rabuffetti M Sipio E Di Diverio M Moroni I. Electromyographic and biomechanical analysis of step negotiation in charcot marie tooth subjects whose level walk is not impaired. Gait Posture. (2018) 62:497–504. 10.1016/j.gaitpost.2018.04.014 29679921

[53] Ardestani MM Kinnaird CR Henderson CE Hornby TG. Compensation or recovery? Altered kinetics and neuromuscular synergies following high-intensity stepping training poststroke. Neurorehabil Neural Repair. (2019) 33:47–58. 10.1177/1545968318817825 30595090 PMC12761792

[54] Fung J Barbeau H. A dynamic EMG profile index to quantify muscular activation disorder in spastic paretic gait. Electroencephalogr Clin Neurophysiol. (1989) 73:233–44. 10.1016/0013-4694(89)90124-7 2475328

[55] Den Otter AR Geurts ACH Mulder T Duysens J. Abnormalities in the temporal patterning of lower extremity muscle activity in hemiparetic gait. Gait Posture. (2007) 25:342–52. 10.1016/j.gaitpost.2006.04.007 16750632

[56] Dobkin BH. Strategies for stroke rehabilitation. Lancet Neurol. (2004) 3:528–36. 10.1016/S1474-4422(04)00851-8 15324721 PMC4164204

